# Dose-Response Effects of Epichlorohydrin on Male Reproductive Function in Rats

**DOI:** 10.5487/TR.2009.25.4.203

**Published:** 2009-12-30

**Authors:** Kang-Hyeon Kim, In-Sik Shin, Jeong-Hyeon Lim, Sung-Hwan Kim, Na-Hyeong Park, Changjong Moon, Sung-Ho Kim, Dong-Ho Shin, Jong-Choon Kim

**Affiliations:** 16 Animal Medical Center, College of Veterinary Medicine, Chonnam National University, Gwangju, 500-757 Korea; 26 Department of Veterinary Medicine, Chonnam National University, Gwangju, 500-757 Korea

**Keywords:** Epichlorohydrin, Reproductive dysfunction, Sperm, Histopathology, Rats

## Abstract

Present study was conducted to investigate potential effects of epichlorohydrin on testicular and epididymal function in male rats. The test chemical was administered to adult male rats by gavage at dose levels of 0, 3.125, 12.5, and 50 mg/kg/day for 7 days. Testicular and epididymal function were assessed by measurement of reproductive organ weight, testicular spermatid count, epididymal sperm count, motility and morphology, and histopathology in rats. At 50 mg/kg, a decrease in the sperm motility and an increase in the incidence of sperm abnormalities were observed. Histopatho-logical examinations revealed an increase in the incidence of histopathological changes including cell debris in the ducts, vacuolization of the epithelial cells, oligospermia, and epithelial disruption in the proximal caput epididymidis. At 12.5 mg/kg, an increase in the incidence of histopathological changes of the epididymidis was found. There were no treatment-related effects at 3.125 mg/kg. These results show that 7-day repeated oral administration of epichlorohydrin to male rats results in adverse effects on sperm motility, sperm morphology, and epididymal histology at ≥ 12.5 mg/kg/day.

## INTRODUCTION

Epichlorohydrin (ECH) is a colorless liquid with an irritating and chloroform-like odor that is used for the manufacture of epoxy resins, glycerin, coatings, adhesives, paints, varnishes, insecticides, and many other products. Due to its increased production and wide-spread use, the potential risk caused by ECH in humans has steadily increased, which may result in severe health impacts (Giri, 1970; SRI, 2007). Exposure to ECH can occur by inhalation, ingestion, and eye or skin contact in the general population, as well as in workers with specific occupations. ECH is rapidly and extensively absorbed following ingestion, inhalation, and dermal contact (Gingell *et al.*, 1985).

The toxicity of ECH has been extensively studied in short and long term animal studies over the past several decades. Previous studies demonstrated that ECH is an anti-fertility agent that acts both as an epididymal toxicant and an agent capable of directly affecting sperm motility (Cooper *et al.*, 1974; Hahn, 1970; van Esch and Wester, 1982). A single dose greater than 50 mg/kg produces reduced fertility and large retention cysts in the ductuli efferentes and proximal caput of the epididymidis in male rats (Cooper *et al.*, 1974). Repeated or prolonged exposure at levels greater than 6.25 mg/kg/day can cause decreased sperm motility and sterility in male rats (Toth *et al.*, 1989; van Esch and Wester, 1982). Although it has been suggested that the anti-fertility effects of ECH maybe resulted from reduced sperm motility and sperm metabolism, potential effects of ECH on male reproductive function including spermatogenesis and spermiogenesis has not been fully elucidated.

The purpose of the present study was to evaluate potential effects of ECH on testicular and epididymal function after 7-day repeated oral administration in male rats. Although the most likely route of exposure is by inhalation, the oral route of exposure was selected for this study because there is a possibility of oral absorption.

## MATERIALS AND METHODS

***Animals.*** Ten-week old male Sprague-Dawley rats were purchased from Orient-Bio Inc. (Seoul, Korea) and used after one week of quarantine and acclimatization. The animals were maintained in a room at 23 ± 3°C, with a relative humidity of 50 ± 10%, under a controlled 12 h light/dark cycle. All rats were housed two per cage in stainless wire cages and were allowed sterilized tap water by ultraviolet (UV) irradiation and fed on commercial rodent chow (Samyang Feed Co, Wonju, Korea) ad libitum. The Institutional Animal Care and Use Committee of Chonnam National University approved the protocols for the animal study, and the animals were cared for in accordance with the Guidelines for Animal Experiments of Chonnam National University.

***Test chemical and treatment.*** ECH was purchased from Aldrich (Milwaukee, WI, USA). The test chemical was dissolved in corn oil (Sigma-Aldrich, St. Louis, MO, USA) and were freshly prepared daily before the treatment. The daily application volume (10 ml/kg body weight) of ECH was calculated in advance based on the most recently recorded body weight of the individual animal. ECH was administered orally to rats for 7 days. The vehicle control rats received an equivalent volume of corn oil alone.

***Experimental groups and dose selection.*** Healthy males were assigned randomly into four experimental groups of 6 rats each: three treatment groups receiving 3.125, 12.5, and 50 mg/kg/day ECH and a vehicle control group. The doses selected are on the basis of toxicity studies as described earlier (Cooper *et al.*, 1974; Toth *et al.*, 1989; van Esch and Wester, 1982).

***Observation of animals.*** All rats were observed daily for clinical sings throughout the treatment period and abnormal signs were recorded individually for type, observation day/time and duration. Body weights were measured at the initiation of treatment and on days 1, 2, and 7 of test.

***Necropsy and organ weight.*** The animals were sacrificed 24 h after the last administration using anesthetic ether. Complete gross postmortem examinations were performed on all terminated animals. The gross weights of the testis, epididymis, prostate gland, and seminal vesicle were measured and then their weights relative to body weight calculated.

***Sperm examination.*** Sperm analysis was conducted as previously described (Chung *et al.*, 2006; Kim *et al.*, 1999). At the scheduled necropsy, the testes and epididymides were removed and weighed. The left testis was homogenized with 12 ml of distilled water for the sperm head counts. The sperm suspension was placed into a hemacytometer (Neubauer, Germany) and the number of spermatids was counted using a light microscope (Leica, Germany). The left cauda epididymis was homogenized with 10 ml physiological saline to determine the sperm counts. The number of sperm was counted as in the testis. For the motility measurements, the sperm was obtained from the ductus deferens, placed in Hanks' balanced salt solution (pH 7.2, Sigma Chemical Co., St. Louis, MO, USA) containing 5 mg/ml bovine serum albumin (Sigma Chemical Co., St. Louis, MO, USA) and maintained at 37°C. Motility was observed using a microscope with a stage warmer (Microwarm plat; MDF-10, Japan). The sperm morphology was also examined using optical microscopy of the sperm smears (sperm suspension containing 1% Eosin Y) collected from the left ductus deferens. Two hundred sperm per animal were evaluated for head and tail defects by light microscope. The sperm morphology was classified as normal, small head, amorphous head, two heads/tails, straight hook, excessive hook, folded tail, short tail, and no tail.

***Histopathological examinations.*** The right testis and right epididymidis were fixed with Bouin fixative and 10% neutral buffered formalin solution, respectively. The tissues were routinely processed, embedded in paraffin and sectioned at 3~5 μm. These sections were stained with Hematoxylin-Eosin for histopathologic examination and then examined microscopically.

***Statistical analyses.*** All values are expressed as mean ± SD. Variables such as body weights, organ weights, and spermatid count were subjected to one-way ANOVA, and Dunnett multiple comparison test (Dunnett, 1964) was conducted when analytic results were significant. The percentages of motile sperm and sperm abnormalities were analyzed by the Kruskal-Wallis nonparametric ANOVA (Kruskal and Wallis, 1952), followed by multiple comparisons using Dunnett's test. The clinical signs, necropsy findings and histopathological findings were represented as frequencies and were subjected to the Fisher's exact probability test (Fisher, 1970) when necessary. The difference was considered statistically significant when $p < 0.05$ or 0.01.

## RESULTS AND DISCUSSION

During the study period, no treatment-related mortality was noted in animals exposed to ECH at any dose tested. However, the male rats of the high dose group showed treatment-related occurrence of clinical signs such as nasal discharge ($n = 3$), soft feces ($n = 2$), reduced locomotor activity ($n = 2$), and piloerection ($n = 2$). These findings may be attributed to mucus and gastrointestinal irritation effects of ECH because ECH is a chemical that acts as an eye, skin, and nasal irritant (Gage, 1959). It was previously reported that acute respiratory irritation with hemorrhages and severe edema occurs in rats after inhalation or oral application (Kremneva and Tolgskaja, 1961; Laskin *et al*., 1980). As presented in Tables 1 and 2, no statistically significant change in body weight and organ weight, compared to the control group, was observed in all treatment groups.

As shown in Table 3, epididymal sperm motility in the 50 mg/kg group was significantly decreased, while sperm morphological abnormalities in the group were significantly increased in comparison to the control group. These results obtained in this study were consistent with the results of previous studies. Toth *et al.* (1991) demonstrated that a 23-day repeated oral dose of ECH to male rats can reduce the percent of motile sperm at doses ≥ 6.25 mg/kg, and decrease curvilinear velocity and straight-line velocity at doses ≥ 12.5 mg/kg using computer-assisted sperm motion analysis. Slott *et al.* (1990) also reported that inhaled epichlorohydrin at

**Table 1.** Body weights of male rats treated with epichlorohydrin for 7 days

| Items | Epichlorohydrin (mg/kg/day) | | | |
|---|---|---|---|---|
| | 0 | 3.125 | 12.5 | 50 |
| No. of males | 6 | 6 | 6 | 6 |
| Day 0 | 338.3 ± 15.57[a] | 337.5 ± 13.85 | 336.9 ± 12.38 | 338.0 ± 11.44 |
| Day 1 | 358.7 ± 12.79 | 355.9 ± 14.20 | 356.8 ± 19.87 | 358.1 ± 10.35 |
| Day 2 | 368.8 ± 10.47 | 366.9 ± 15.44 | 359.8 ± 17.31 | 362.9 ± 17.53 |
| Day 7 | 375.4 ± 12.07 | 372.1 ± 12.43 | 361.8 ± 13.71 | 365.3 ± 9.95 |

[a]Values are presented as means ± SD.

**Table 2.** Relative organ weights of male rats treated with epichlorohydrin for 7 days

| Items | Epichlorohydrin (mg/kg/day) | | | |
|---|---|---|---|---|
| | 0 | 3.125 | 12.5 | 50 |
| No. of male rats | 6 | 6 | 6 | 6 |
| Body weight (g) | 375.4 ± 12.07[a] | 372.1 ± 12.43 | 361.8 ± 13.71 | 365.3 ± 9.95 |
| Testis: left (%) | 0.49 ± 0.054 | 0.49 ± 0.052 | 0.50 ± 0.034 | 0.53 ± 0.041 |
| right (%) | 0.48 ± 0.052 | 0.49 ± 0.051 | 0.50 ± 0.040 | 0.51 ± 0.029 |
| Epididymis: left (%) | 0.16 ± 0.017 | 0.16 ± 0.016 | 0.17 ± 0.004 | 0.17 ± 0.019 |
| right (%) | 0.16 ± 0.017 | 0.16 ± 0.018 | 0.16 ± 0.006 | 0.16 ± 0.016 |
| Seminal vesicles (%) | 0.27 ± 0.042 | 0.27 ± 0.040 | 0.29 ± 0.048 | 0.27 ± 0.049 |
| Prostate (%) | 0.15 ± 0.019 | 0.15 ± 0.020 | 0.17 ± 0.027 | 0.15 ± 0.021 |

[a]Values are presented as means ± SD.

**Table 3.** Sperm analysis of male rats treated with epichlorohydrin for 7 days

| Items | Epichlorohydrin (mg/kg/day) | | | |
|---|---|---|---|---|
| | 0 | 3.125 | 12.5 | 50 |
| No. of male rats | 6 | 6 | 6 | 6 |
| Sperm heads in testis (× $10^6$) | 234.5 ± 28.71[a] | 241.5 ± 20.56 | 230.0 ± 15.14 | 234.9 ± 12.41 |
| Sperm in caudal epididymis (× $10^6$) | 155.1 ± 23.41 | 162.1 ± 21.08 | 160.1 ± 13.24 | 160.0 ± 22.50 |
| Sperm motility (%) | 81.4 ± 9.91 | 82.3 ± 10.07 | 68.8 ± 6.30 | 55.0 ± 11.63** |
| Sperm abnormalities (%) | 7.6 ± 3.51 | 7.3 ± 4.17 | 10.0 ± 4.06 | 15.7 ± 4.01** |
| Small head | 0.0 ± 0.00 | 0.0 ± 0.00 | 0.0 ± 0.00 | 0.6 ± 0.71 |
| Amorphous head | 1.2 ± 1.30 | 1.4 ± 1.52 | 1.6 ± 0.89 | 2.7 ± 2.83 |
| Two heads/tails | 0.8 ± 0.84 | 0.2 ± 0.45 | 0.6 ± 0.89 | 0.5 ± 0.58 |
| Straight hook | 1.8 ± 2.49 | 0.6 ± 0.89 | 0.0 ± 0.00 | 1.8 ± 1.50 |
| Folded tail | 2.4 ± 1.14 | 2.8 ± 1.92 | 3.6 ± 3.51 | 5.8 ± 2.22 |
| Short tail | 0.0 ± 0.00 | 0.0 ± 0.00 | 0.0 ± 0.00 | 0.3 ± 0.50 |
| No tail | 1.4 ± 1.14 | 2.3 ± 1.81 | 4.2 ± 1.79 | 4.0 ± 2.83 |

[a]Values are presented as means ± SD.
**$p < 0.01$ compared with the control group.

**Table 4.** The incidence and severity of histopathological lesions in epididymis of male rats treated with epichlorohydrin for 7 days

| Findings | Grade | Epichlorohydrin (mg/kg/day) | | | |
|---|---|---|---|---|---|
| | | 0 | 3.125 | 12.5 | 50 |
| Cell debris in duct | - | 6 | 6 | 2 | 0 |
| | + | 0 | 0 | 4 | 3 |
| | ++ | 0 | 0 | 0 | 3 |
| | +++ | 0 | 0 | 0 | 0 |
| Vacuolization of epithelial cell | - | 6 | 6 | 2 | 0 |
| | + | 0 | 0 | 4 | 6 |
| | ++ | 0 | 0 | 0 | 0 |
| | +++ | 0 | 0 | 0 | 0 |
| Oligospermia | - | 6 | 6 | 3 | 0 |
| | + | 0 | 0 | 3 | 5 |
| | ++ | 0 | 0 | 0 | 1 |
| | +++ | 0 | 0 | 0 | 0 |
| Epithelial disruption | - | 6 | 6 | 2 | 1 |
| | + | 0 | 0 | 4 | 2 |
| | ++ | 0 | 0 | 0 | 3 |
| | +++ | 0 | 0 | 0 | 0 |

Grades are as follows: - (normal), + (mild), ++ (moderate), and +++ (severe).

100 ppm produced specific, transient decreases in rat sperm velocity. Cassidy *et al.* (1983) reported that a single oral dose of ECH at 50 mg/kg causes an increase in the incidence of abnormal sperm in male rats.

The results of the histopathological examination are summarized in Table 4. Histopathological changes were observed only in the proximal caput epididymis at dose levels of ≥ 12.5 mg/kg. Characteristic histopathologic findings observed in the present study included cell debris in the ducts, vacuolization of the epithelial cells, oligospermia, and epithelial disruption in the epididymidis (Fig. 1B). According to the previous studies, Hahn (1970) reported that male Sprague-Dawley rats dosed orally with 15 mg/kg/day for 12 days showed loss of fertility within the first week of exposure, but no effects were seen on copulatory behavior or on the histopathology of the reproductive organs. John *et al.* (1983) also reported that inhalation exposure of ECH to male rats for 10 weeks resulted in a dose-related infertility at 93.4 and 189.0 $mg/m^3$ air, but microscopic examination did not reveal any abnormalities in the reproductive organs. On the contrary, Cooper *et al.* (1974) reported that five oral doses of 20 mg/kg or one single oral dose of 100 mg/kg produces large retention cysts in the ductuli efferentes and proximal caput of the epididymidis in male rats. Jelks *et al.* (2001) and Jelks and Miller (2001) also demonstrated that a single oral dose of alpha-chlorohydrin, a metabolite of ECH, at ≥ 25 mg/kg results in white

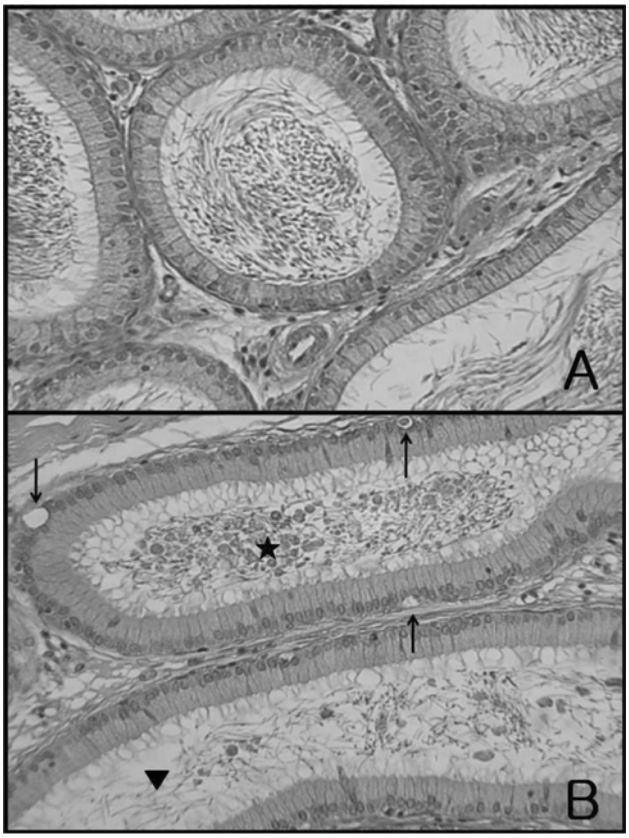

**Fig. 1.** Representative photographs of the epididymis sections from the control and high dose groups stained with Hematoxylin & Eosin. (A) Control rat caput epididymis. The epithelium and luminal contents look normal. (B) Caput epididymis treated with 50 mg/kg/day ECH, with cell debris in ducts (★), oligospermia (▼), and vacuolization of epithelial cells (↑).

pustule, sperm granuloma formation, sloughed epithelial cells in the lumen, and epithelial cell vacuolization and disruption in the rat epididymidis. The results of above studies and the present study clearly show that ECH and its metabolite have adverse effects on epididymal histology in rats.

In conclusion, the 7-day repeated oral dose of ECH to rats caused an increase in the incidence of histopathological changes of the epididymidis at 12.5 mg/kg/day; and a decrease in the sperm motility and an increase in the incidence of sperm abnormalities and histopathological changes of the epididymidis at 50 mg/kg/day.

## ACKNOWLEDGEMENTS

This work was supported by the Grant of the Korean Ministry of Education, Science and Technology (The Regional Core Research Program/Biohousing Research

Institute). This work was also supported by the Grant of the Animal Medical Center, Chonnam National University.
